# Wearable sensors based on colloidal nanocrystals

**DOI:** 10.1186/s40580-019-0180-7

**Published:** 2019-04-02

**Authors:** Woo Seok Lee, Sanghyun Jeon, Soong Ju Oh

**Affiliations:** Department of Materials Science and Engineering, Korea University, Seoul, 02841 Republic of Korea

**Keywords:** Nanocrystals, Strain sensors, Temperature sensors, Pressure sensors, Wearable devices

## Abstract

In recent times, wearable sensors have attracted significant attention in various research fields and industries. The rapid growth of the wearable sensor related research and industry has led to the development of new devices and advanced applications such as bio-integrated devices, wearable health care systems, soft robotics, and electronic skins, among others. Nanocrystals (NCs) are promising building blocks for the design of novel wearable sensors, due to their solution processability and tunable properties. In this paper, an overview of NC synthesis, NC thin film fabrication, and the functionalization of NCs for wearable applications (strain sensors, pressure sensors, and temperature sensors) are provided. The recent development of NC-based strain, pressure, and temperature sensors is reviewed, and a discussion on their strategies and operating principles is presented. Finally, the current limitations of NC-based wearable sensors are discussed, in addition to methods to overcome these limitations.

## Introduction

With the rapid development of the internet of things (IoT), wearable electronic devices have attracted significant attention in research fields and industry, as they can be used for remote health care monitoring and human–machine interfaces [1–5]. They are commonly integrated into clothes, glasses, and watches, and directly attached to human skin to collect physical, chemical, and biological signals generated by humans or their surroundings [6, 7]. Among the various components of wearable devices, strain, pressure, and temperature sensors are critical for the monitoring of human motion, health or physiological information, and external stimuli [8–13]. Significant research effort was directed toward the enhancement of the performance of the abovementioned wearable sensors using various materials such as graphene, carbon nanotubes, organic materials, and silicon nanomembranes; and/or by designing unique device structures [14–17]. However, costly and complex high temperature and/or high vacuum processes such as sputtering, reactive-ion etching, and thermal deposition are generally required to synthesize functional materials and/or manufacture devices [18–22]. This results in a high production cost, which limits their commercialization.

Colloidal nanocrystals (NCs) are considered promising building blocks for the next generation of wearable sensors, as they provide the following advantages. First, NCs can be synthesized at a large scale using wet chemical methods, and the resulting NC inks can be deposited onto various substrates in a large area under room-temperature and in an atmospheric environment using a solution based process such as roll-to-roll printing, drop casting, spin-coating, and inkjet printing [23–30]. Second, the electronic, optical, and magnetic properties of NCs can be easily controlled by adjusting their size, shape, composition, and surface state; thus enabling them to demonstrate application-specific functionality [31–37]. Based on these advantages, significant research effort has been directed toward the realization of high performance NC-based strain, pressure, and temperature sensors by the control of the interparticle distance between the NCs, or by the design of new NC structures [38–47].

In this brief review, the ligand exchange strategy of NCs for the development of conductive and functional NC thin films with application-specific properties for strain, pressure, and temperature sensors is discussed. Thereafter, a summary on the recently reported NC-based strain, pressure, and temperature sensors is presented, in addition to a brief explanation of their strategies, operating principles, and practical applications. Moreover, the review includes an overview of the current challenges, and a perspective on the future methods for the realization of advanced NC-based wearable sensors.

## Review

### Surface ligand exchange of NCs for specific applications

**Fig. 1:**
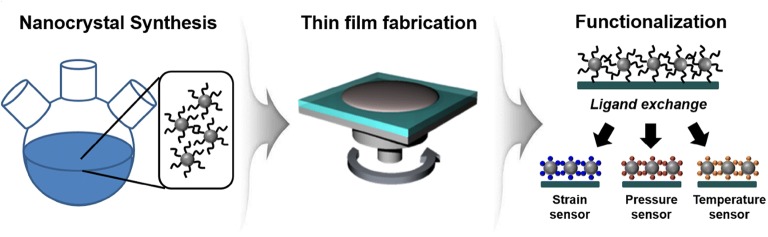
Schematic of the synthesis, thin film fabrication, and surface ligand exchange processes of NCs

Nanocrystals (NCs) are composed of hundreds to thousands of atoms with diameters smaller than 100 nm [27]. Moreover, NCs are generally synthesized with long organic chains such as oleic acid and oleylamine as their surface capping ligands, using wet chemical methods [27]. These long organic ligands control the size and shape of the NCs during the synthesis, and enable the dispersion of the NCs in organic solvents after the synthesis and washing procedures [27, 28]. The resulting NC inks allow for the formation of NC thin films on various substrates using solution-based process such as spin-coating, drop casting, inkjet printing, and roll-to-roll printing [32]. The as-synthesized NC thin films are electrical insulators, given that long original ligands result in long interparticle distances, which limit the efficient charge transport and effective coupling between each NC. Thus, a ligand exchange strategy is generally used to improve the electrical properties and provide functionality [24, 32]. The original long ligands are replaced with short organic or inorganic ligands by immersing the as-synthesized NC thin films into a ligand exchange solution. It is common knowledge that the conductivity of NC thin films varies from 10^12^ to 10^−6^ Ω cm depending on the lengths of the surface ligands, which determine the interparticle distance [48–50]. In addition, the overall property of NCs is governed by their surface ligand chemistry, due to their high-surface-to-volume ratio [32, 48]. Therefore, application-specific properties can be realized by selecting appropriate types of surface ligands for the ligand exchange process. This enables NCs with the same composition to be used as active materials for different applications such as strain, pressure, and temperature sensors by adjusting their surface chemistry through the ligand exchange process (Fig. 1) [49–51].

### NC-based strain sensors

**Fig. 2:**
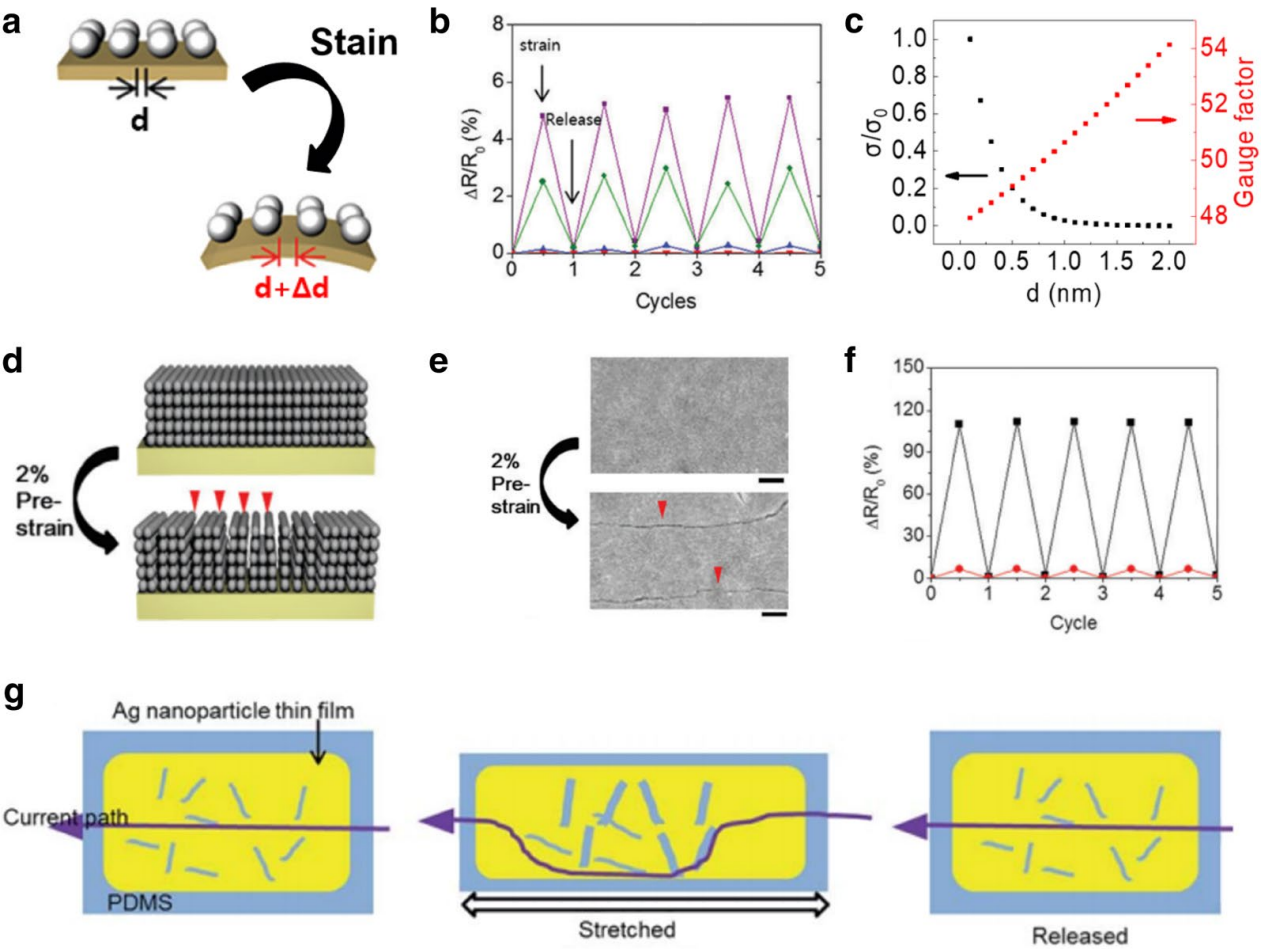
**a** Schematic of NC thin films after applied strain. **b** Cycle tests with application of 0.2% strain on MPA—(purple), EDT—(green), Cl—(red), and Br—(blue) treated Ag NC thin films. **c** Conductivity and gauge factor of NC thin films as a function of initial interparticle distance. **d** Schematics of crack formation strategy. **e** SEM images of Ag NC thin films after crack formation. **f** Cycle tests with application of 0.2% strain on MPA-treated Ag NC thin films before (red) and after (black) crack formation and **g** schematic of current path change of Ag NC thin films with cracks after the strain application (Figure reproduced from **a**, **b**, and **d**–**f** [50], Copyright 2017, Royal Society of Chemistry; **c** [59], Copyright 2017, Wiley-VCH; **g** [60] Copyright 2014, Royal Society of Chemistry)

Strain sensors are devices that measure the electromechanical deformation of objects. Strain gauge sensors are key components in wearable electronic devices, as they are able to measure the breathing rate, heartbeat, and pulse for applications in wearable health care systems; in addition to a wide range of human motion, for human–machine interfaces [8–10]. The sensitivity of strain sensors is referred to as the gauge factor, which is defined by the following equation:

1 $$ {\text{G}} = \left( {\Delta R/R_{0}  } \right)/\varepsilon $$

where ΔR is the change in resistance, R_0_ is the base resistance, and ε is the applied strain. Commercial strain sensors based on metal thin films have a limited gauge factor of ~ 3 [52]. In contrast, NC-based strain sensors have larger gauge factors, which are higher than 10 [39]. This is attributed to the unique hopping or tunneling transport mechanism in NC thin films, which can be expressed by the following equation:

2 $$ \upsigma =\upsigma_{0} { \exp }\left( { -\upbeta{\text{d}}} \right){ \exp }\left( { - \frac{{E_{a} }}{\text{kT}}} \right) $$

where σ_o_ is the intrinsic conductivity, β is the tunneling decay constant, d is the interparticle distance, k is the Boltzmann constant, T is the temperature, and E_a_ is the activation energy. Thus, the external strain increases the interparticle distance and exponentially decreases the conductance of NC thin films, according to the Eq. (2) (Fig. 2a). The hopping or tunneling transport mechanism promotes the sensitivity of the NC thin films to the applied strain, and results in a high gauge factor, when compared with conventional metal thin films [44, 45]. Significant effort has been directed toward the enhancement of the sensitivity of NC thin films by adjusting the parameters in Eq. (2). First, several studies were conducted on the effects of controlling the composition of NCs using pure metals, metal alloys, metal oxides, and semiconducting materials [39, 40, 50]. Second, several researchers adjusted the size, shape, and morphology of NCs to improve the sensitivity [53–56]. Third, surface ligand modification using various inorganic or organic ligands was carried out to control the interparticle distance, tunneling decay term, and activation energy between the NCs [53, 57]. Lee et al. investigated the electrical and electromechanical properties of Ag NC thin films with respect to the type of surface ligands (Fig. 2b) [50]. The long carbonic ligands of the as-synthesized Ag NC thin films were replaced with short inorganic ligands of ammonium chloride (NH_4_Cl) and tetrabutylammonium bromide (TBAB), and short organic ligands of 3-mercaptopropionic acid (MPA) and 1,2-ethanedithiol (EDT). The NH_4_Cl- and TBAB-treated Ag NC thin films exhibited a significant decrease in the resistivity (10^−5^ Ω cm) and notably low gauge factor of ~ 1, given that the short inorganic ligand treatment resulted in a minimal interparticle distance or even touch between each NC. In contrast, the MPA- and EDT-treated Ag NC thin films exhibited a relatively high resistivity of over 1 Ω cm, and high gauge factor of ~ 30. Although NC-based strain sensors have higher gauge factors when compared with those of typical metal thin film based strain gauges; the gauge factors are excessively low for the detection of subtle bio-signals, which limits their use in advanced applications such as bio-integrated devices [58].

There is a theoretical limit to the gauge factor of NC thin films, which is predicted by Eq. (2) [59]. Although the gauge factor can be improved by increasing the initial interparticle distance or the tunneling decay term, the initial conductivity of the NC thin films decreases exponentially, which limits the practical applications of NC based strain sensors (Fig. 2c). To overcome this intrinsic limitation of NC thin films, novel strategies such as artificial crack formation and a NC heterostructure design were developed [50, 59]. Lee et al. introduced artificial nanocracks into MPA-treated Ag NC thin films by the application of a high pre-strain to the NC thin films (Fig. 2d, e) [50]. The external strain opens closed cracks, which results in a significant increase in resistance. Using this approach, a high gauge factor of over 300 was achieved after the crack formation (Fig. 2f). Lee et al. also demonstrated Ag NC thin films with micro-crack based strain sensors, which exhibit a high stretchability, durability, stability, and sensitivity (Fig. 2g) [60].

**Fig. 3:**
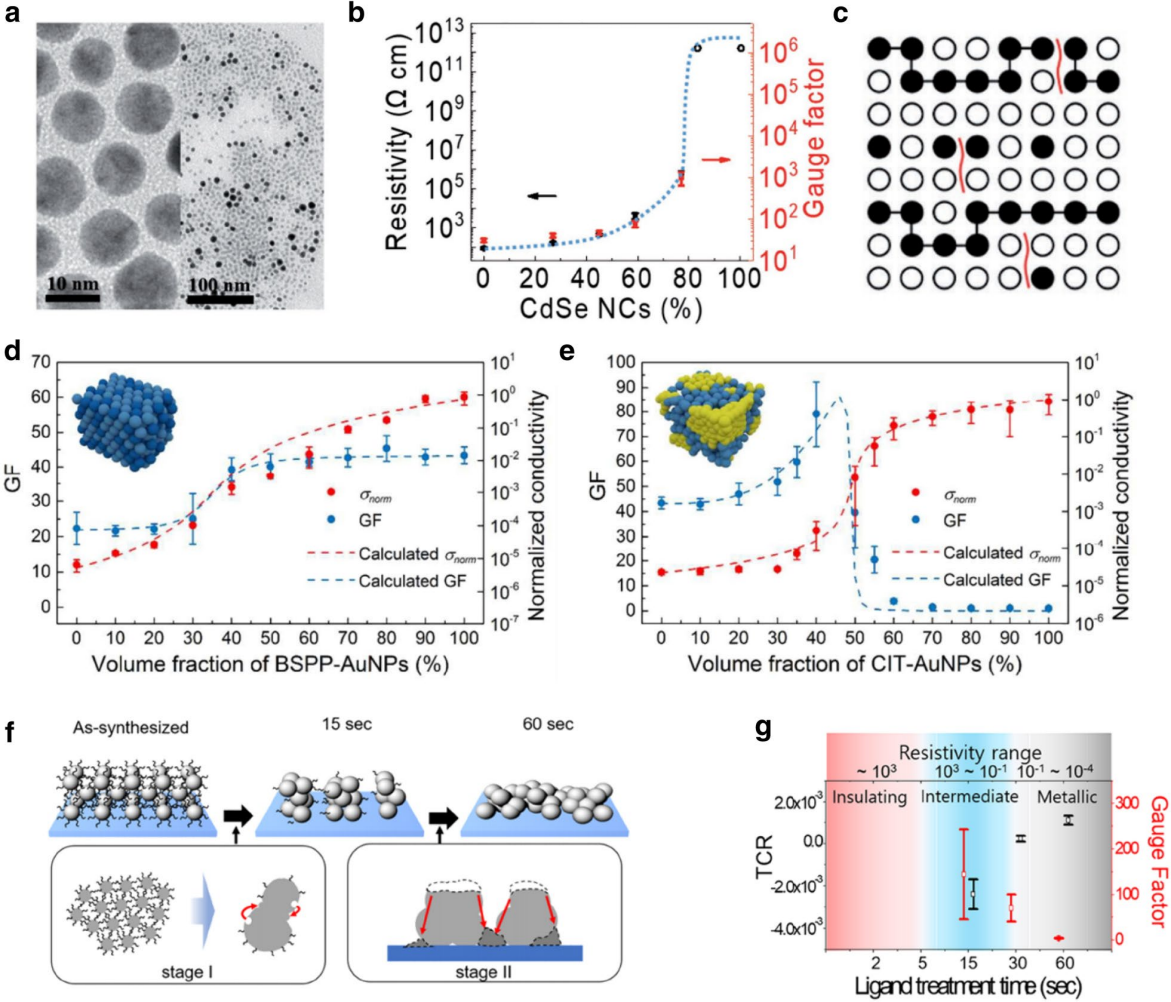
**a** TEM images of (left) pure Au NC and (right) Au-CdSe hybrid NC thin films. **b** Resistivity and gauge factor of Au-CdSe NC hybrid thin films with cracks depending on the fraction of CdSe NCs. **c** Schematic of square lattice structures for Au-CdSe NC hybrid thin films with cracks according to the site and bond percolation model. The conductivities and gauge factor of **d** homogeneous arrangement shell binary NC materials (SBNM) and **e** heterogeneous arrangement SBNM. **f** Schematic of structural transformation of NC thin films during ligand exchange. **g** TCR and gauge factor of Ag NC thin films depending on ligand exchange time (Figure reproduced from **a**–**c** [59], Copyright 2017, Wiley-VCH; **d**, **e** [61], Copyright 2018, Royal Society of Chemistry; **f**, **g** [64], Copyright 2018, American Chemical Society)

Besides the crack formation strategy, a percolation strategy was developed to improve the performance of NC-based strain sensors, with respect to the sensitivity, by the design of a metal–insulator hetero-NC structure [59, 61]. The metal–insulator structure exhibited a unique electrical resistance behavior, which was dependent on the ratio of the metal to the insulator, according to the percolation theory [62]. In particular, the conductivity increases significantly as the ratio of metallic components approaches the percolation threshold where external perturbation such as strain can induce significant changes in resistance [63]. Lee et al. designed the metal–insulator structure based strain sensors using Au and CdSe NCs as metallic and insulating components, respectively (Fig. 3a) [59]. The resistivity and gauge factor increased as the ratio of insulating components of the CdSe NCs increased in the heterostructure (Fig. 3b). Artificial nanocracks were created in the NC heterostructure to further enhance the sensitivity, thus achieving a high gauge factor of over 1000. To clarify the origin of the high gauge factor in hetero-NC thin films with cracks, the site and bond percolation theory was developed by considering Au and CdSe NCs as occupied and empty sites, and by bridging ligands of EDT and open cracks as connected and disconnected bonds, respectively (Fig. 3c).

Zhang et al. designed homogeneous and heterogeneous arrays of NCs with different surface capping ligands, and then evaluated their electrical and electromechanical properties (Fig. 3d, e) [61]. As demonstrated, the gauge factor of hybrid structures can be tuned from 1 to 96 by adjusting the volume ratio of each NC according to the percolation theory. Lee et al. implemented the partial ligand exchange strategy to induce cracks and to simultaneously design NC thin films in a metal–insulator transition state for strain sensor applications (Fig. 3f) [64]. The conventional ligand exchange process was conducted with sufficient treatment time for the formation of fully ligand exchanged functional NC thin films. In the case of Ag NC thin films treated with TBAB for over 60 s, fully ligand exchanged, highly conductive, and strain-insensitive NC thin films were formed. In contrast, partially ligand exchanged Ag NC thin films with naturally formed cracks were observed when the as-synthesized Ag NC thin films were treated with TBAB for 15 s, which exhibited an intermediate conductivity and high gauge factor of up to 300 (Fig. 3g).

**Fig. 4:**
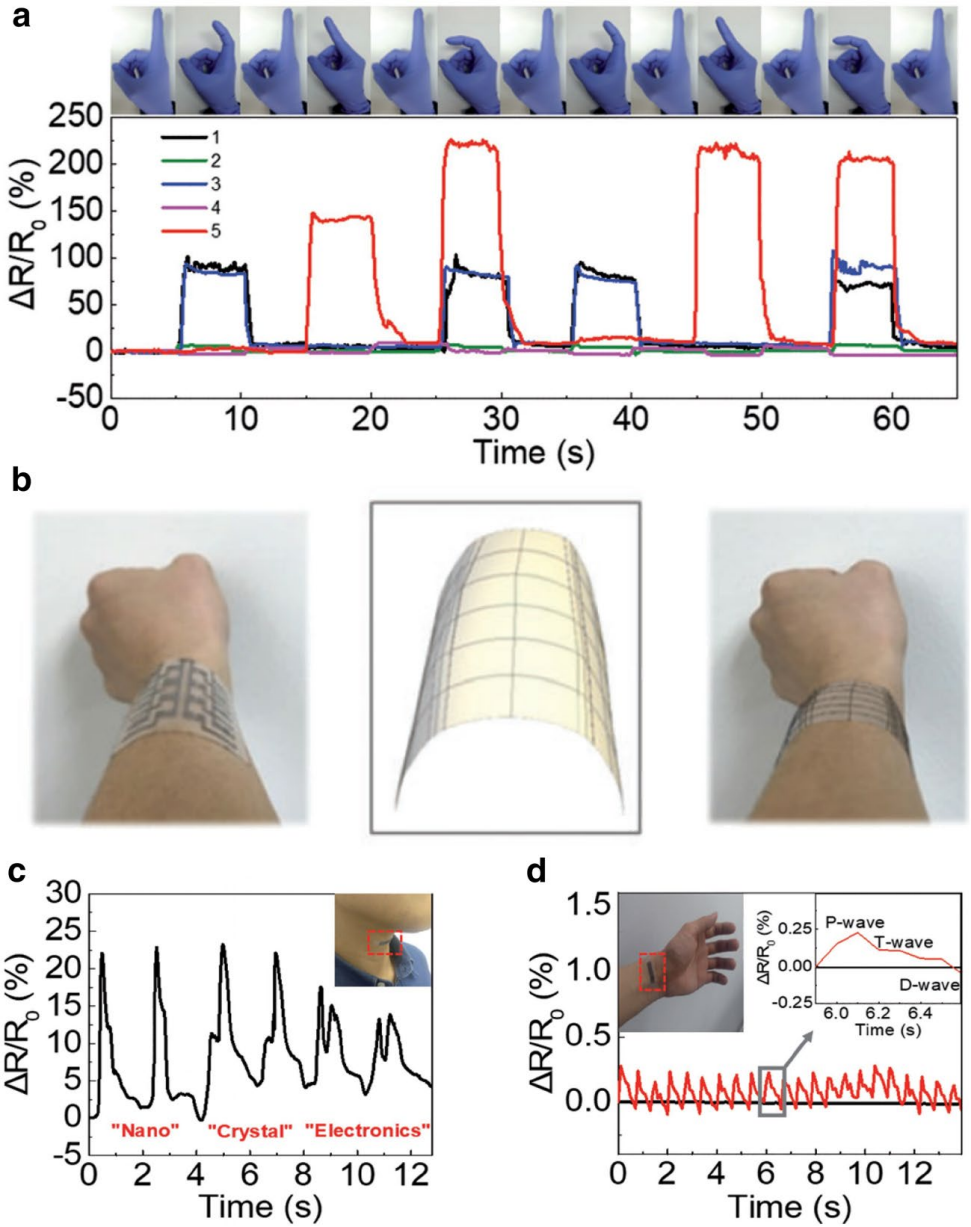
Applications of NC-based strain sensors for **a** human motion detection, **b** human body design, **c** voice recognition, and **d** pulse monitoring (Figure reproduced from **a**, **c**, and **d** [59], Copyright 2017, Wiley-VCH; **b** [50], Copyright 2017, Royal Society of Chemistry)

Based on the advantages of solution processible materials, in addition to their high sensitivity, NC-based strain sensors can be used for various practical applications. Figure 4a presents the detection results for different finger bending motions using NC-based strain sensors. Figure 4b illustrates that NC-based strain sensors can be used to design human body by measuring the curvature of a human arm. Moreover, NC-based strain sensors can be used for voice recognition (Fig. 4c). By attaching sensors to a human neck and measuring the resistance of the sensors with respect to the movement of the vocal cords, the words spoken by a person wearing the sensors can be distinguished. Another potential application of NC-based strain sensors is wearable health care monitoring. Figure 4d illustrates that NC-based sensors attached to a human wrist can measure the pulse in real time.

### NC-based pressure sensors

A pressure sensor is a device that detects a force applied to a specific area. Moreover, it is one of the most important mechanical sensors, in addition to strain gauges. Pressure sensors have attracted considerable attention in various research fields, as they can be used for medical diagnoses, touch screen, health care monitoring, and industrial applications [65–68]. Among the various types of pressure sensors, capacitive or resistive type pressure sensors, which convert applied pressure to electrical signals, are the most efficient and cost-effective [69, 70]. Significant effort was directed toward the improvement of the performance of pressure sensors, such as their stretchability, sensitivity, durability, reliability, linearity, and detection range using various materials, and by the development of unique device architectures [11, 71, 72]. In particular, nanoscale/microscale bumpy structures such as pyramids or hemispheres are generally used to improve the sensitivity and enlarge the detection range of pressure sensors [11, 21, 73]. However, complex and toxic process such as e-beam lithography or chemical etching are generally required for the fabrication of these structures.

**Fig. 5:**
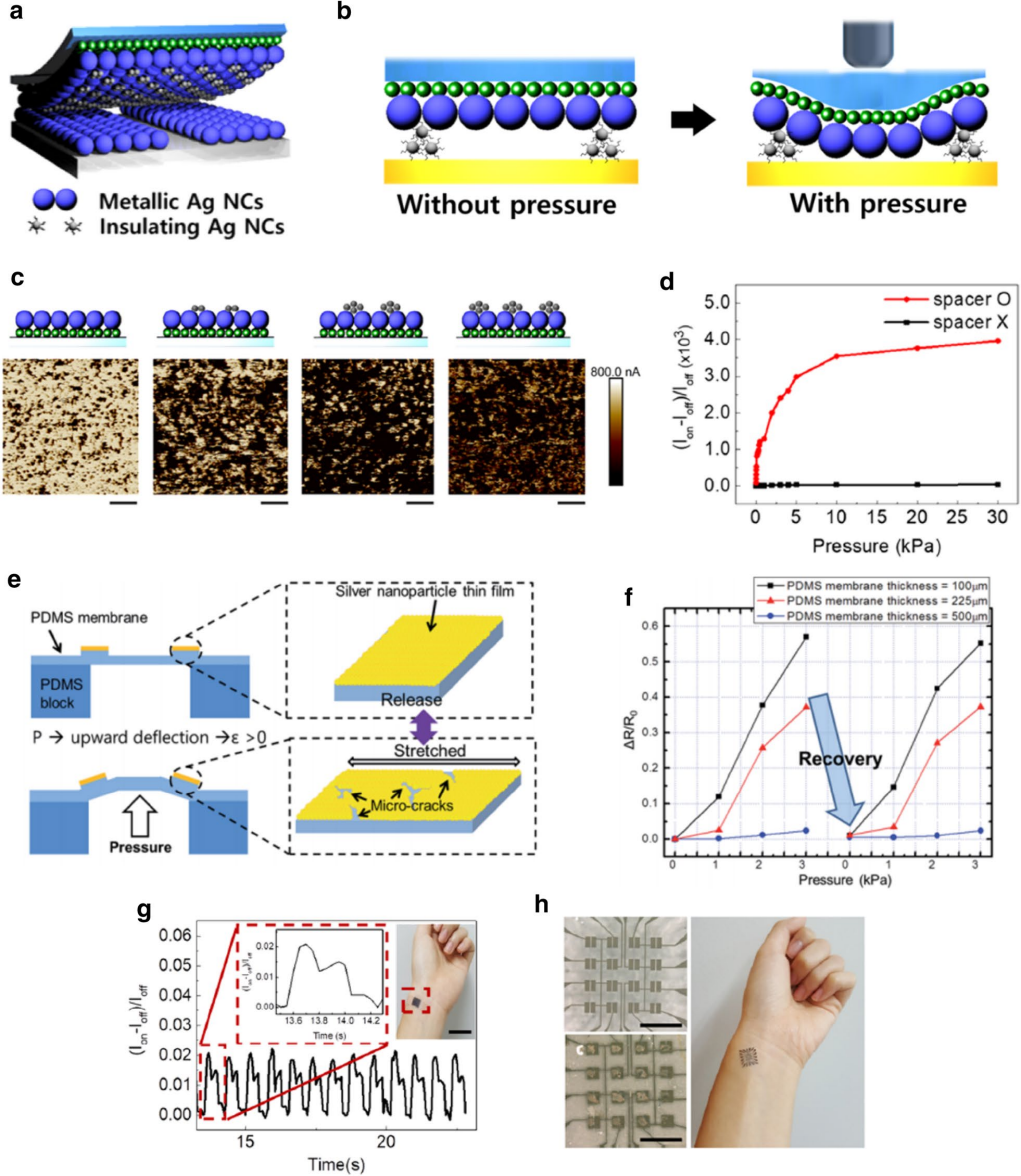
**a** Schematic and **b** operating principles of hybrid NC-based pressure sensors. **c** C-AFM profile of hybrid Ag NC thin films with respect to the amount of as-synthesized Ag NC thin films. **d** Relative current change of hybrid Ag NC-based pressure sensors with respect to the applied pressure. **e** Schematic of NC-based pressure sensors with cracks and their operating principles. **f** Relative change in resistance of NC-based pressure sensors with cracks after the application of pressure. **g** Real-time pulse monitoring using NC-based pressure sensors. **h** Images of high-pixel tactile NC-based pressure sensors (Figure reproduced from **a**–**d**, **g**, and **h** [51], Copyright 2018, American Chemical Society; **e**, **f** [60], Copyright 2014, Royal Society of Chemistry)

Kim et al. designed these nanoscale/microscale structures using Ag NCs by controlling their surface chemistry and developing unique hybrid NC structures using a solution process (Fig. 5a) [51]. The pressure sensor consists of top and bottom electrodes. The bottom electrodes were pre-patterned with a separation gap of 1 mm between two conductive electrodes. The top electrodes were designed as a hybrid metal–insulator structure made of conductive NH_4_Cl-treated Ag NC thin films and insulating as-synthesized Ag NC thin films. The insulating NC thin films acted as a spacer between the top and bottom electrodes, thus limiting the contact between the two electrodes without pressure (Fig. 5b). A new contact point was formed and/or the existing contact area was enlarged, which increased the conductance of the pressure sensors with respect to the magnitude of the applied pressure. By optimizing the thickness and uniformness of the as-synthesized Ag NC thin films on conductive NH_4_Cl-treated Ag NC thin films, a sensitivity of over 500 kPa^−1^ and wide pressure detection range of 0.01–100 kPa were achieved (Fig. 5c, d).

Lee et al. demonstrated flexible pressure sensors based on NC thin films with micro-cracks (Fig. 5e) [60]. Pressure applied to the bottom of the devices induced positive strain on the NC films with cracks, which increased the resistance. It was revealed that the sensitivity and pressure detection range can be adjusted by controlling the thickness of the substrates (Fig. 5f).

High performance NC-based pressure sensors can be utilized in various applications. The practicality and functionality of NC-based pressure sensors are demonstrated in applications that involve pulse monitoring and tactile sensors (Fig. 5g, h).

### NC-based temperature sensors

There is a continuous increase in the demand for high performance temperature sensors, given that accurate temperature measurement is very important in several industries, medical fields, and research fields. The recent advancement of wearable technology promotes the rapid development of wearable temperature sensors, as they are essential components of wearable devices for health care monitoring or disease diagnoses based on body temperature measurements [13, 14]. Several studies were conducted to improve the sensitivity, stability, and durability of wearable temperature sensors, and to enlarge their temperature detection range using carbon materials, polymers, and thin metal films [74, 75]. However, complex multi-step procedures, which include high temperature and high vacuum processes, are mostly used for the fabrication of the wearable temperature sensors.

NCs can be synthesized at a large scale and deposited onto various substrates with at low-cost using solution based processes [27, 32]. Thus, to develop cost-effective and highly sensitive NC-based temperature sensors, the temperature-dependent electrical characterization of NC thin films was investigated by several researchers [38, 49, 76].

**Fig. 6:**
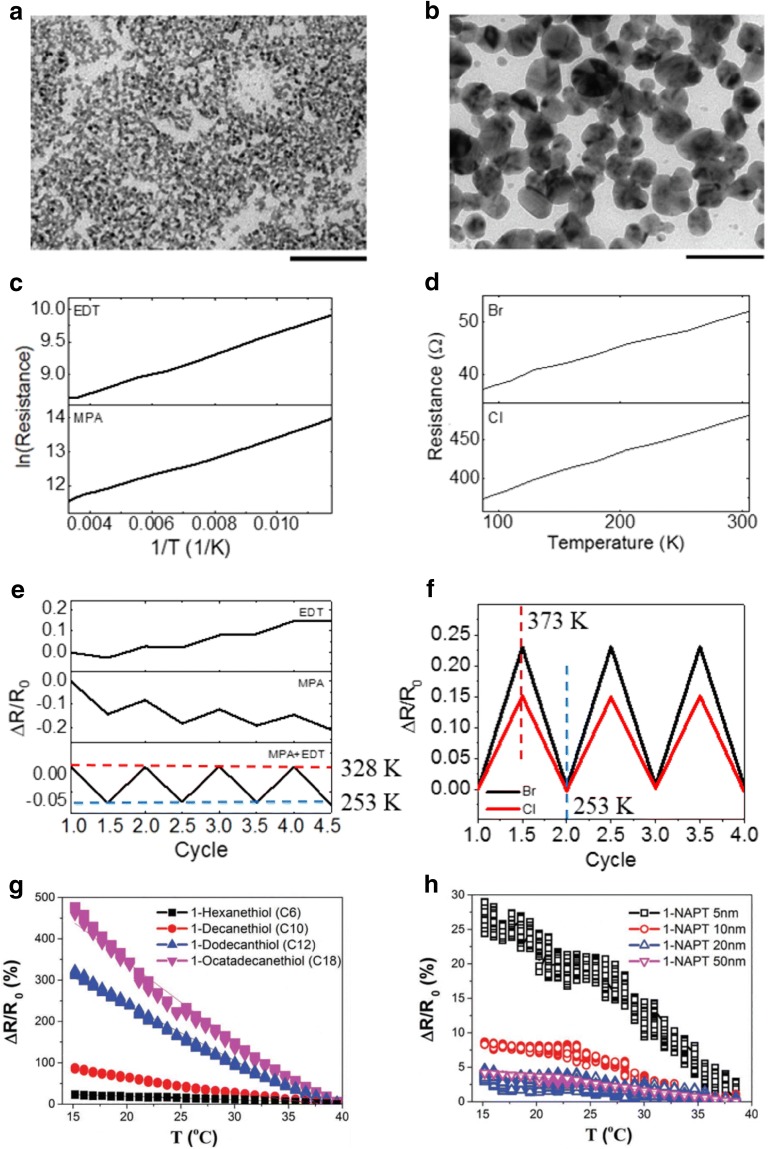
TEM images of **a** EDT- and **b** TBAB-treated Ag NC thin films (scale bar: 100 nm). **c** Arrhenius plot of resistance and temperature of (top) EDT- and (bottom) MPA-treated Ag NC thin films. **d** Change in resistance of (top) TBAB- and (bottom) NH_4_Cl-treated Ag NC thin films with respect to temperature. **e** Change in resistance of (top) EDT-, (middle) MPA-, and (bottom) MPA + EDT-treated Ag NC thin films during temperature cycle tests. **f** Change in resistance of (red) TBAB- and (blue) NH_4_Cl-treated Ag NC thin films during temperature cycle tests. Temperature-dependent electrical behavior of Au NC thin films with respect to the **g** type of surface ligands and **h** NC size (Figure reproduced from **a**–**f** [49], Copyright 2017, Wiley-VCH; **g**, **h** [76], Copyright 2017, Wiley-VCH)

Joh et al. evaluated the electrical properties of Ag NC thin films with respect to temperature by engineering the surface chemistry using ligand exchange methods [49]. The Ag NC thin films exhibited different charge transport mechanisms that were dependent on the surface ligand of the NCs. Organic ligands such as MPA or EDT for the capped Ag NC thin films exhibited interparticle distances of approximately 1 nm and followed the hopping transport mechanism, as presented in Eq. (2) (Fig. 6a). From the combination of Eqs. (1) and (2) and Ohm’s law, the following equation was obtained for the change in resistance as a function of the temperature and strain:

3 $$ \frac{\Delta R}{{R_{0} }} = e^{{\left( { - {\raise0.7ex\hbox{${E_{a} }$} \!\mathord{\left/ {\vphantom {{E_{a} } {k_{B} }}}\right.\kern-0pt} \!\lower0.7ex\hbox{${k_{B} }$}}\Delta \left( {{\raise0.7ex\hbox{$1$} \!\mathord{\left/ {\vphantom {1 T}}\right.\kern-0pt} \!\lower0.7ex\hbox{$T$}}} \right)} \right)}} e^{{\left( {G\varepsilon } \right)}} - 1 $$

In contrast, when the as-synthesized Ag NC thin films were treated with short inorganic ligands, such as NH_4_Cl and TBAB, a significant decrease in the interparticle distance and active sintering of adjacent NCs were observed (Fig. 6b). The sintered Ag NCs allow charge carriers to move with a metallic or band transport behavior, which is expressed by following equation:

4 $$ \frac{\Delta R}{{R_{0} }} = \alpha \Delta T + {\text{G}}\upvarepsilon $$

where α is a temperature coefficient of resistance (TCR).

Furthermore, the MPA- or EDT-treated Ag NC thin films exhibited a negative resistance change (negative TCR) as the strain increased, whereas a positive resistance change was observed for the NH_4_Cl- or TBAB-treated Ag NC thin films with positive TCR values of 1.03 × 10^−3^ K^−1^ and 1.34 × 10^−3^ K^−1^, respectively (Fig. 6c–f).

Segev-Bar et al. investigated the temperature-dependent electrical properties of Au NC thin films with respect to their size and organic surface ligands. As observed, the temperature sensitivity increased as the NC size and length of the surface ligands increased (Fig. 6g, h) [76].

**Fig. 7:**
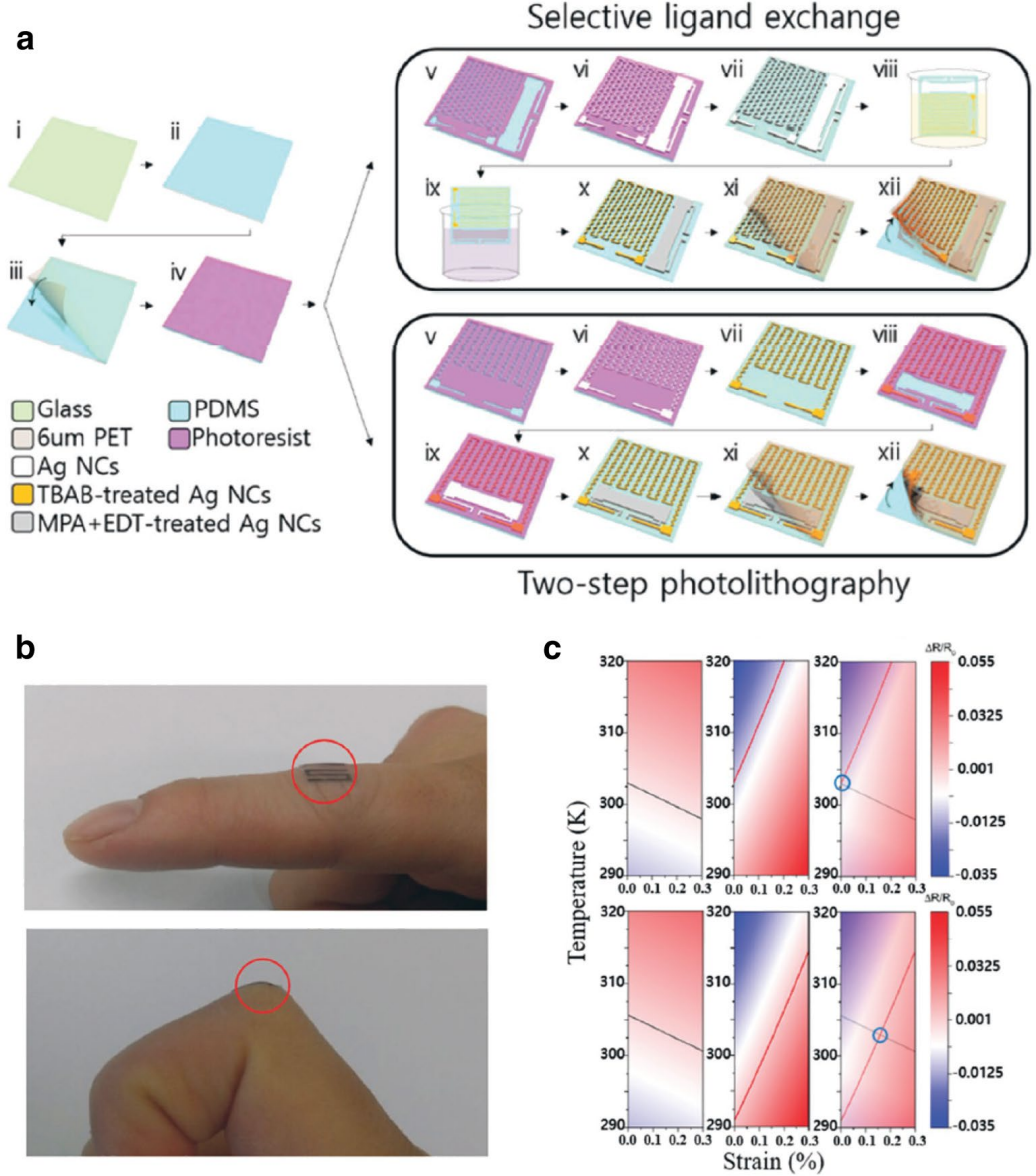
**a** Schematic of fabrication process for the strain–temperature sensors. **b** Images of the sensor on human finger (top) in a flat state and (bottom) in a bent state. **c** Simulation and experimental results of the sensors (top) in a flat state and (bottom) bent state (Figure reproduced from **a–c** [49], Copyright 2017, Wiley-VCH)

The de-coupling of strain and temperature is critical for wearable temperature sensors, as the changes in resistance may be due to changes in the strain and/or temperature [67, 77]. For an accurate measurement of the real body temperature, the effect of the strain during natural body movement on the change in resistance should be neglected based on the fundamental understanding of the charge transport in active materials with respect to the changes in the strain and temperature, in addition to the unique device structure. Unfortunately, as predicted by Eqs. (3) and (4), the resistance of the NC thin films treated with organic and inorganic ligands is influenced by the strain, in addition to the temperature. This limits the accuracy of the body temperature measurement when NC-based sensors are attached to human skin, owing to the strain generated during body movement. Joh et al. solve this problem by integrating MPA + EDT- and TBAB-treated Ag NC thin films into a single device using a solution-based process (Fig. 7a) [49]. Given that the MPA + EDT- and TBAB-treated Ag NC thin films have negative and positive TCRs, respectively, in addition to different gauge factors; the temperature and strain can be measured simultaneously by solving Eqs. (3) and (4). For verification, the relative changes in resistance of the strain–temperature sensor mounted on the human finger were observed and compared with the theoretical values (Fig. 7b, c). The real temperature of the finger measured using an infrared (IR) sensor was 303.6–303.4 K, and the strain calculated using the bending radius of the finger was approximately 0.16%. The temperature and strain measured using the Ag NC-based temperature-strain sensors were 303.15 K and 0.162%, respectively, which confirms the high sensitivity and accuracy of the sensors.

## Conclusion and perspective

Wearable electronics have attracted significant attention, as they can be utilized in remote health care systems, human–machine interfaces, and soft-robotics, among other applications. NCs can overcome the limitations of conventional wearable devices due to their solution processability and tunable properties. Based on these advantages, significant research effort has been directed toward the improvement of the performance of NC-based wearable sensors (strain, pressure, and temperature sensors), as discussed above. However, the NC-based wearable sensors can be further improved. First, conformal contact with human skin is a critical requirement for wearable electronics, for the efficient and accurate detection of human signals [78, 79]. It is therefore necessary to design NC-based wearable sensors using soft elastomers that have stiffnesses similar to that of human skin. Moreover, the effects of the strain, pressure, and temperature on the wearable sensors should be considered, given that all these external perturbations could induce changes in the resistance of wearable sensors [49, 67, 76]. For example, changes in the applied pressure and temperature can modify the resistance of strain sensors, thus limiting the accuracy of the real strain measurement. Therefore, novel methods to decouple unwanted stimuli should be developed for the realization of NC-based wearable sensors with high accuracies. Furthermore, a power supply should be considered, to fully realize NC-based skin-mountable wearable sensors, given that conventional heavy and bulky batteries cannot be used in the system [80, 81]. Hence, self-powered NC-based wearable sensors should be developed to realize the next generation of wearable technology. Finally, while considerable achievements in printing and patterning methods have been demonstrated such as transfer printing, there is still room for improvement in the manufacturing of NC-based wearable sensors [82]. For example, multiple steps of mask alignment, light exposure, and development using photoresists are generally required in conventional patterning methods. To reduce the fabrication steps and costs, advanced patterning techniques such as direct optical lithography using light-responsive ligands without photoresists should be developed for the realization of practical and cost-efficient NC-based wearable devices [83].
